# Severe combined emphysematous infections of the urinary tract in a diabetic patient: A case report

**DOI:** 10.1016/j.radcr.2026.02.044

**Published:** 2026-03-19

**Authors:** Saleck Choumad, Aichetou Mohamed el hacen, Khattary Oumar, Ahmed Ebedda, Itimad Nassar, Kaoutar Imrani

**Affiliations:** Department of Central Radiology, UHC Ibn Sina Mohammed V University, Rabat, Morocco

**Keywords:** Emphysematous pyelonephritis, Emphysematous cystitis, Diabet, Prostatic abscess, Pelvic abscess, CT imaging

## Abstract

Emphysematous infections of the urinary tract are rare but life-threatening conditions, most commonly affecting diabetic and immunocompromised patients. We report a case of a 55-year-old male with uncontrolled type 2 diabetes and chronic alcoholism who presented in septic shock. CT imaging revealed emphysematous pyelonephritis, emphysematous cystitis, a pelvic abscess in the recto-vesical pouch, and bilateral periurethral prostatic abscesses. The case illustrates the importance of early imaging, prompt diagnosis, and multidisciplinary management in preventing fatal outcomes.

## Introduction

Emphysematous urinary tract infections (EUTIs) are rare gas-forming infections primarily affecting diabetic and immunocompromised patients [1,2]. The main forms include emphysematous pyelonephritis (EPN) and emphysematous cystitis (EC) [3]. Although individually reported, their simultaneous occurrence with associated pelvic and prostatic abscesses is uncommon. CT imaging plays a pivotal role in defining the extent of disease and guiding management.

## Case report

A 55-year-old man with poorly controlled type 2 diabetes and chronic alcoholism was brought to the emergency department for acute confusion and hypotension. On admission, he was febrile (39.4°C), tachycardic, hypotensive, and had a Glasgow Coma Scale score of 12/15. Clinical examination revealed suprapubic tenderness.

Laboratory tests showed marked inflammatory syndrome, acute renal failure, severe hyperglycemia, and elevated lactate levels. Urinalysis demonstrated pyuria, positive nitrites, and bacteriuria. Blood and urine cultures grew extended-spectrum beta-lactamase–producing *Escherichia coli*. A contrast-enhanced abdominopelvic CT scan was performed.

CT revealed bilaterally enlarged kidneys with multiple gas locules in the right calyceal system, consistent with EPN, associated with renal hypoenhancement and perirenal fat stranding. The bladder wall was diffusely thickened, with intraluminal and intramural gas foci adherent to the anterior and posterior walls, consistent with EC.

**Fig. 1:**
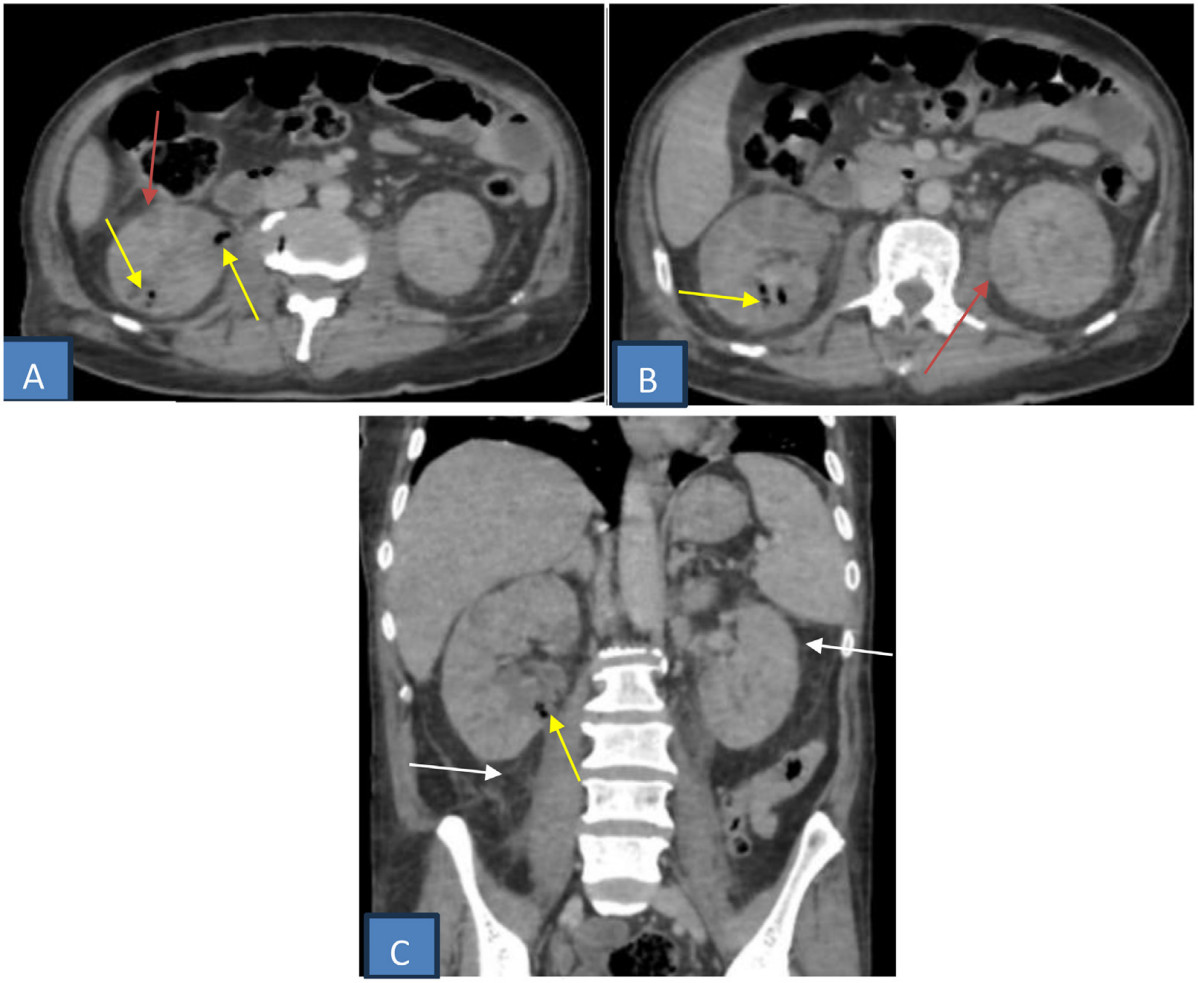
Axial (A and B) and coronal (C) contrast-enhanced CT scan showing an enlarged right kidney with multiple gas locules in the calyceal system (yellow arrow), consistent with emphysematous pyelonephritis. Bilateral renal parenchymal hypoenhancement (red arrow) and perirenal fat stranding (white arrow).

**Fig. 2:**
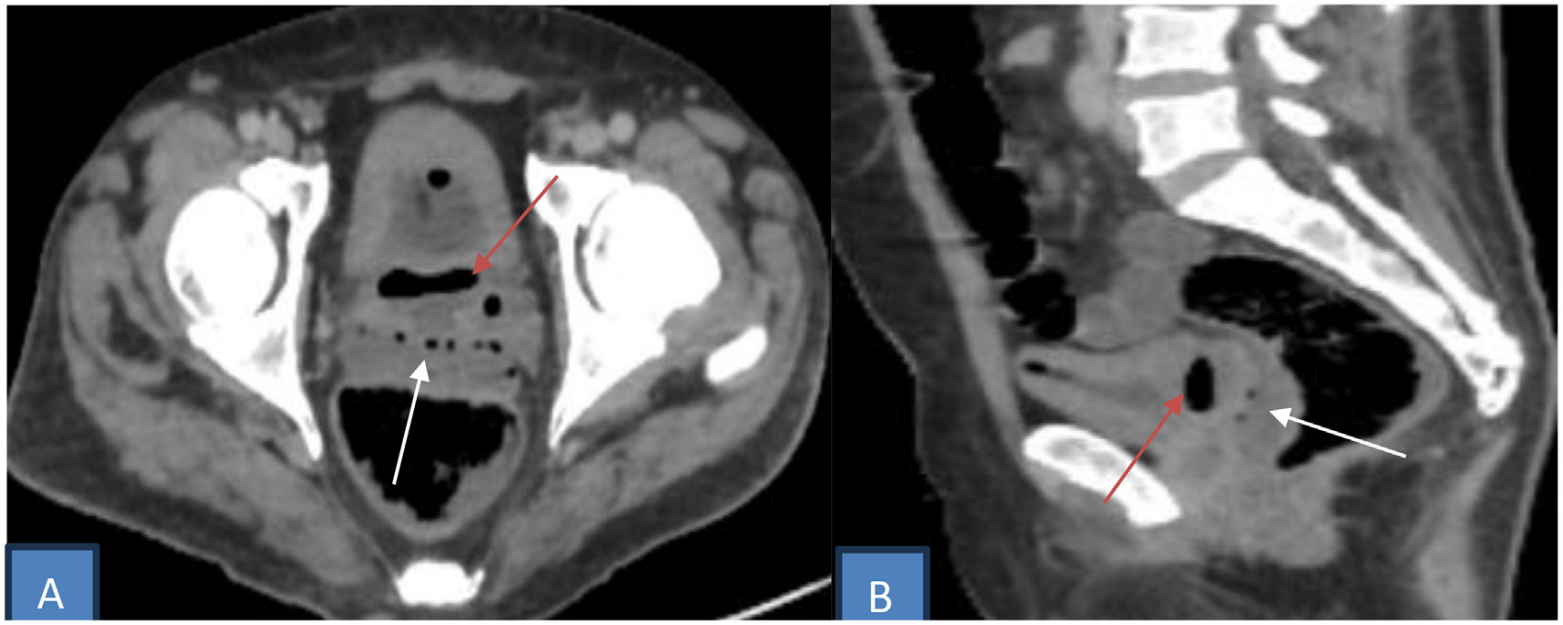
Axial (A) and sagittal (B) pelvic CT image showing marked thickening of both the anterior and posterior bladder walls. Gas bubble adherent to the anterior wall, with another in the posterior bladder wall (red arrow), consistent with emphysematous cystitis. A pelvic abscess containing air in the rectovesical pouch (white arrow) suggesting bladder rupture.

**Fig. 3:**
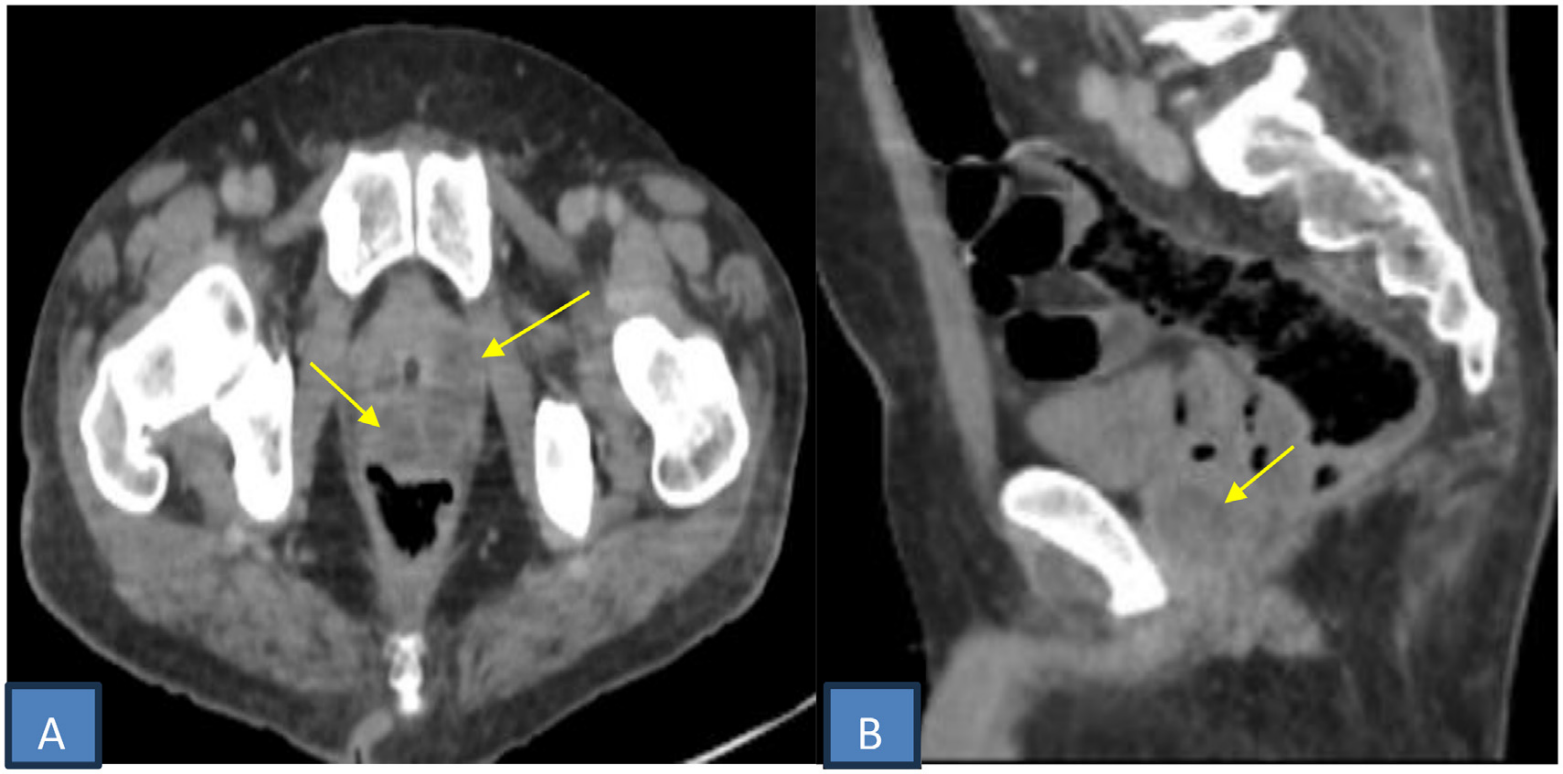
Axial (A) and sagittal (B) pelvic CT images showing two periurethral fluid collections with thick enhancing walls and no internal gas (arrow), consistent with prostatic abscesses.

A pelvic abscess containing gas was identified in the rectovesical pouch. Two periurethral collections surrounding the prostatic urethra showed peripheral enhancement without internal gas, compatible with prostatic abscesses (Fig. 1, Fig. 2, Fig. 3).

Delayed post-contrast acquisitions showed no opacification of the excretory pathways, likely reflecting severe renal impairment.

The patient was admitted to the intensive care unit and treated with aggressive fluid resuscitation, carbapenem-based antibiotic therapy guided by culture results, vasopressor support, and strict glycemic control. CT-guided drainage of the pelvic abscess was successfully performed, while the prostatic abscesses were managed conservatively.

Clinical status progressively improved, with normalization of inflammatory markers and renal function.

A follow-up contrast-enhanced CT performed three weeks later demonstrated complete resolution of intramural bladder gas and marked regression of the pelvic abscess. No bladder diverticulum or fistulous tract was identified, and bladder wall thickness had normalized.

## Discussion

EUTIs are rare gas-producing infections typically observed in immunocompromised hosts, particularly patients with diabetes mellitus, which remains the most significant risk factor [4]. These infections are usually caused by gas-forming organisms, such as *E. coli, Klebsiella pneumoniae, Proteus mirabilis*, and *Clostridium* species [5,6]. In diabetic patients, high tissue glucose levels, impaired vascular perfusion, and defective immune responses facilitate bacterial fermentation and gas production within the urinary tract [7].

Radiologic evaluation, especially CT, plays a central role in both diagnosis, and staging of emphysematous infections. CT allows reliable differentiation between EC, characterized by gas within or surrounding the bladder wall, and EPN, defined by gas within the renal parenchyma or perinephric space [8]. Ultrasound may reveal echogenic foci with posterior dirty shadowing, but its sensitivity is lower than CT [9]. CT also enables identification of complications such as abscess formation, extension of gas beyond anatomical compartments, and possible vascular involvement. The Huang and Tseng classification for EPN remains widely used to guide therapeutic decision-making [10].

The simultaneous occurrence of EPN and EC is uncommon. Previous series report concurrent involvement in approximately 10%-20% of EUTIs, particularly in poorly controlled diabetic patients.

The differential diagnosis of intraluminal or parietal gas within the urinary tract includes infectious, iatrogenic, inflammatory, and neoplastic causes. Enterovesical fistulas, particularly in patients with diverticulitis or colorectal cancer, may mimic EC; the presence of adjacent colonic wall thickening or contrast leakage into the bladder helps establish the diagnosis [11]. Post-instrumentation intravesical gas is typically transient and not associated with bladder wall thickening or clinical signs of infection [12]. Radiation cystitis and intravesical tumors, including squamous cell carcinoma, may also present with bladder wall irregularity or necrosis, potentially mimicking EC [13].

Regarding prostatic collections, differential diagnoses include benign prostatic hyperplasia, prostatitis, and prostate carcinoma. However, the presence of central necrosis, peripheral enhancement, and systemic sepsis strongly favors prostatic abscess [14].

In the present case, delayed imaging demonstrated no bladder diverticulum or fistulous communication, and intramural gas was confined to the bladder wall, supporting the diagnosis of EC rather than diverticular abscess.

Complications of emphysematous urinary infections are numerous and potentially life-threatening. In EPN, they include renal infarction, perirenal abscess, septic thrombophlebitis, and systemic sepsis with multiorgan failure [15]. Delayed recognition or treatment may result in nephrectomy or death. EC may lead to bladder wall necrosis, perforation, or pelvic cellulitis if infection spreads through fascial planes [16].

In our case, the additional findings of pelvic abscess and prostatic abscesses illustrate rare but severe extensions of infection. Prostatic abscesses may rupture into the urethra, rectum, or perineum, requiring prompt drainage [17]. Pelvic abscesses, particularly in the rectovesical pouch, may extend retroperitoneally or involve intra-abdominal compartments if inadequately treated [18].

Management depends on both imaging findings and clinical severity. Broad-spectrum antibiotics targeting Gram-negative and anaerobic organisms must be initiated promptly, and strict glycemic control is essential in diabetic patients. Abscesses often require image-guided drainage. In severe or refractory EPN, particularly Huang class 3 or 4, nephrectomy may be required. Multidisciplinary management involving radiologists, urologists, and intensivists improves outcomes in complex presentations, such as this one.

## Conclusion

This case highlights the diagnostic value of CT in detecting life-threatening urinary tract infections. Prompt imaging led to early diagnosis and initiation of targeted management, including antibiotics, glycemic control, and abscess drainage. Multidisciplinary care is essential to improve outcomes in such complex presentations.

## Authors' contributions

All authors contributed to the implementation and realization of this work. All authors declare that they have read and approved the final version of this manuscript.

## Patient consent

Written informed consent was obtained from the patient(s) for their anonymized information to be published in this article.
